# Psychometric Limitations of the Center for Epidemiologic Studies-Depression Scale for Assessing Depressive Symptoms among Adults with HIV/AIDS: A Rasch Analysis

**DOI:** 10.1155/2016/2824595

**Published:** 2016-03-03

**Authors:** Caryl L. Gay, Anders Kottorp, Anners Lerdal, Kathryn A. Lee

**Affiliations:** ^1^Department of Family Health Care Nursing, University of California San Francisco, San Francisco, CA 94143, USA; ^2^Department of Research, Lovisenberg Diakonale Hospital, 0440 Oslo, Norway; ^3^Department of Occupational Therapy, College of Applied Health Sciences, University of Illinois at Chicago, Chicago, IL 60612, USA; ^4^Division of Occupational Therapy, Department of Neurobiology, Care Sciences and Society, Karolinska Institutet, 14183 Huddinge, Sweden; ^5^Department of Nursing Science, Institute of Health and Society, Faculty of Medicine, University of Oslo, 0318 Oslo, Norway

## Abstract

The Center for Epidemiological Studies-Depression (CES-D) scale is a widely used measure of depressive symptoms, but its psychometric properties have not been adequately evaluated among adults with HIV/AIDS. This study used an item response theory approach (Rasch analysis) to evaluate the CES-D's validity and reliability in relation to key demographic and clinical variables in adults with HIV/AIDS. A convenience sample of 347 adults with HIV/AIDS (231 males, 93 females, and 23 transgenders; age range 22–77 years) completed the CES-D. A Rasch model application was used to analyze the CES-D's rating scale functioning, internal scale validity, person-response validity, person-separation validity, internal consistency, differential item functioning (DIF), and differential test functioning. CES-D scores were generally high and associated with several demographic and clinical variables. The CES-D distinguished 3 distinct levels of depression and had acceptable internal consistency but lacked unidimensionality, five items demonstrated poor fit to the model, 15% of the respondents demonstrated poor fit, and eight items demonstrated DIF related to gender, race, or AIDS diagnosis. Removal of misfitting items resulted in minimal improvement in the CES-D's substantive and structural validity. CES-D scores should be interpreted with caution in adults with HIV/AIDS, particularly when comparing scores across gender and racial groups.

## 1. Introduction

Depressive symptoms are common among adults living with HIV or AIDS, with an estimated 20% to 37% having a major depressive disorder [1, 2]. Depression among adults with HIV/AIDS has been associated with high risk behaviors [3], low medication adherence [4], poor health outcomes [5], and reduced quality of life [6, 7]. Accurately and reliably assessing depressive symptoms is critical to research aimed at understanding and treating depression and thereby improving the quality of life of people living with HIV/AIDS.

Although a clinical interview by a trained professional is considered the gold standard for determining a diagnosis of depression based on DSM or ICD criteria, screening instruments and symptom rating scales are often used for research purposes [8]. Symptom rating scales have the benefit of providing a continuous measure of depressive symptom frequency or severity, and screening instruments include a cutoff score indicating the need for further evaluation or a probable diagnosis of depression. Both are typically quick and easy to self-administer.

Of the many different instruments used to assess depressive symptoms, the Center for Epidemiological Studies-Depression (CES-D) scale [9] is one of the more common measures used in HIV/AIDS research [8]. This 20-item self-report instrument has demonstrated good reliability and validity in a variety of populations [10, 11]. Its development was based on Beck's four-factor model of depression, which includes positive affect, negative affect, somatic symptoms and retarded activity, and interpersonal difficulties. While many studies have documented this four-factor structure [12], some research suggests that these factors may vary across different groups [13], which raises concern about its generalizability.

Associations between CES-D scores and demographic factors, such as race/ethnicity [13–15], gender [16], education [17], and income [17–20], have been well documented. However, it is not clear whether these observed differences reflect true group differences, psychometric variability across groups, or a combination of both. Recent studies have begun to address the possibility of racial/ethnic and gender differences in the psychometric properties of the CES-D [14, 21–27], although, to our knowledge, none have addressed the influences of education or income, and none have adequately addressed these issues in the HIV/AIDS population.

For adults with HIV/AIDS, concern has also been raised about the CES-D's inclusion of somatic symptoms of depression, as they may overlap with disease-related symptoms and inflate depression scores among those with HIV/AIDS [28]. Others have shown that the somatic symptoms have little impact on the CES-D's ability to distinguish depressed and nondepressed adults with HIV/AIDS or other chronic conditions [29]. This issue is not limited specifically to HIV disease but has been debated in relation to other diagnostic groups as well [30–33].

Questions have also been raised about the validity of the CES-D's four positive affect items (*felt as good as other people*,* hopeful*,* happy*, and* enjoyed life*), leading some researchers to suggest excluding these items from the total CES-D score [34, 35]. In an early study of inpatients with HIV/AIDS, two of the positive affect items (*felt as good as other people* and* hopeful*) were found not to differ between healthy controls and adults meeting DSM criteria for depression [29]. In the same study, it was also found that the two interpersonal items (*unfriendly *and* felt that people did not like me*) were unable to distinguish nondepressed and depressed patients with HIV/AIDS. How seriously these issues affect the reliability and validity of the CES-D for adults with HIV/AIDS remains unknown.

The psychometric properties of the CES-D have been systematically evaluated using both classical test theory (CTT) and item response theory (IRT) in a variety of populations [34, 36]. Although many studies have specifically evaluated its structural validity using exploratory and confirmatory factor analysis, results have been inconsistent. Furthermore, these approaches are largely limited to the specific sample and cannot determine the degree to which items are equivalent across individuals. IRT approaches, such as Rasch modeling, have certain advantages over CTT and have been used to more fully describe the CES-D's underlying structure, to identify items with poor fit to the rest of the scale, and to identify items that perform inconsistently across groups [30, 37, 38].

Items that fail to perform consistently across groups are said to demonstrate differential item function (DIF). This occurs when a specific item is more or less easily endorsed by certain groups of respondents while controlling for differences in the underlying construct being measured [39]. In the case of the CES-D, DIF occurs when respondents who have the same underlying levels of depression but belong to different subgroups (e.g., gender, race, or health status) have different response patterns to a particular item. This occurs when the location of an item on the depression continuum varies depending on the respondent's group membership. Understanding whether items demonstrate DIF in relation to demographic and clinical variables is critical to interpreting the differences in CES-D scores across various groups [40].

Several studies have used CTT approaches to evaluate the utility of the CES-D for identifying risk of depression among adults with HIV/AIDS [29, 41], but none have used Rasch modeling to evaluate aspects of validity and reliability, including unidimensionality and stability of item function across groups. Therefore, the purpose of this study was to evaluate aspects of the CES-D's validity and reliability using an application of the Rasch model in a sample of adults with HIV/AIDS. Results from this study will also determine whether there is differential item function, or DIF, in relation to several key demographic and clinical variables. Given that a 10-item version of the CES-D has also been suggested for adults with HIV/AIDS [41], we also use the Rasch model to evaluate the psychometric properties of this version in our sample. Although this analysis focuses on adults with HIV/AIDS, the findings may have potential relevance to other adults with chronic illness.

## 2. Method

### 2.1. Participants and Setting

The Symptom and Genetic Study was a prospective longitudinal study aimed at identifying biomarkers of symptom experience among adults with HIV/AIDS [48]. The Committee on Human Research at the University of California, San Francisco (UCSF) approved the study protocol (#10-01357). Participants were recruited from April 2005 to December 2007 using flyers posted at local HIV/AIDS clinics and community sites. Participants provided written informed consent and signed a Health Insurance Portability and Accountability Act release for the use of their protected medical information in research before participation. Study visits, each lasting approximately one hour, were conducted at the University of California, San Francisco, General Clinical Research Center.

Eligible participants were English-speaking adults at least 18 years of age who had been diagnosed with HIV infection at least 30 days before enrollment. To specifically address stable HIV/AIDS-related symptom experience, potential participants were excluded if they currently used illicit drugs (as determined by self-report or by positive urine drug testing prior to the baseline assessment); worked nights (i.e., at least four hours between 12 AM and 6 AM); reported having bipolar disorder, schizophrenia, or dementia; or were pregnant within the prior three months. Participants were not excluded for insomnia but were excluded for other diagnosed sleep disorders, such as apnea and narcolepsy. Research staff conducted eligibility screening by interviewing potential participants in person or by phone.

### 2.2. Measures

#### 2.2.1. Demographic, Clinical, and Laboratory Characteristics

A demographic questionnaire was used to collect information about the participant's age, gender, race/ethnicity, education, and income. A prior diagnosis of AIDS and current medications (including antiretroviral and antidepressants medications) were obtained by self-report. Urine screening was used to detect current illicit drug use both prior to and three days after enrollment. The most recent CD4+ T-cell count and viral load values were obtained from the participant's medical record.

#### 2.2.2. Depressive Symptoms

The Center for Epidemiological Studies-Depression (CES-D) scale [9] was used to assess the frequency of depressive symptoms in the previous week. The CES-D consists of 20 items selected to represent major symptoms in the clinical syndrome of depression. Total scores can range from 0 to 60, with scores of 16 and higher indicating the need for adults to seek clinical evaluation for major depression. The CES-D's four subscales and their range of scores are positive affect (0 to 12), negative affect (0 to 21), somatic symptoms and retarded activity (0 to 21), and interpersonal difficulties (0 to 6). The CES-D has well-established concurrent and construct validity [11, 49–52]. In this study, Cronbach's alpha coefficient for the CES-D was 0.88.

### 2.3. Statistical Analysis

Descriptive statistics were used to summarize the study sample, and nonparametric tests (Kruskal-Wallis and Mann-Whitney *U*) were used to compare the nonnormal distributions of CES-D scores across demographic and clinical groups. A Rasch, partial-credit model application was used to analyze the CES-D scores using Winsteps Rasch analysis software, version 3.69.1.16 [53]. First, the rating scale properties of the original 20-item CES-D were evaluated. A stepwise process was then used whereby items failing to meet standard fit criteria were removed one at a time. If multiple items failed to meet the criterion for a given step, the item with the worst misfit was removed and the step was repeated with the remaining items until all items met the criterion set. Cronbach's alpha coefficient was used as a measure of internal consistency, and principal components analysis (PCA) was used to assess unidimensionality. The analytic approach is summarized in Table 1 and has been previously described [42, 44, 46, 47, 54–56].

## 3. Results

### 3.1. Description of Sample

Of the 560 adults who expressed interest in the study and were screened for eligibility, 116 were not eligible (primarily due to disqualifying psychiatric diagnosis, *n* = 74) and 94 chose not to enroll (by either declining to participate or not showing for the first study visit). Of the 350 adults with HIV/AIDS enrolled in the study, three were excluded from analysis due to missing CES-D data. Thirty participants tested positive for illicit drug use at the second study visit (3 days after enrolling in the study), and their data were retained in the analysis to evaluate DIF related to drug use. Demographic and clinical characteristics of the 347 participants included in the final sample are reported in Table 2. Overall, depressive symptoms were common in this sample, and significant differences in total CES-D scores were observed based on the respondent's gender, education, viral load, antidepressant use, and current illicit drug use. There were also significant demographic and clinical differences in the 4 component scores of the CES-D.

### 3.2. Rating Scale Functioning

The rating scale used in the CES-D met the criteria set. The average measures for each category and thresholds advanced monotonically.

### 3.3. Internal Scale Validity

Of the original 20 items in the CES-D, five failed to demonstrate acceptable goodness-of-fit (see Tables 1 and 3). Through the stepwise process of deleting items with unacceptable goodness-of-fit, items 4 (*felt as good as others*), 8 (*hopeful*), 11 (*restless sleep*), 2 (*poor appetite*), and 16 (*enjoyed life*) were sequentially omitted. The remaining 15 items all demonstrated acceptable goodness-of-fit (range of infit* MnSq* values: 0.65 to 1.30). For subsequent analytic steps, psychometric properties were evaluated for both the original 20-item CES-D and a 15-item version omitting the five misfitting items.

A principal components analysis (PCA) of the residuals, also performed using Winsteps, indicated that the Rasch dimension (depressive symptoms) explained only 32.5% of the variance in the original 20-item CES-D and 37.9% in the 15-item version, which were both below the criterion of ≥50%. The secondary dimension explained 9.4% and 7.4% of the variance in the 20-item and 15-item versions, respectively, both exceeding the set criterion of <5%.

Because these findings failed to support the unidimensionality of the CES-D as a whole, we complemented the analysis of the entire 20-item scale with a PCA of each of the four subscales in order to explore whether they demonstrated higher levels of unidimensionality. Although the variance explained in the subscales was generally higher than for the full scale (either the 20-item or 15-item versions), none of the subscales reached the set criterion of ≥50% explained variance (see Table 4).

### 3.4. Person-Response Validity

In this sample, both the original 20-item CES-D and the 15-item version had an unacceptably high proportion of misfitting respondents (see Table 1), with both exceeding the criterion of <5%. The respondents who demonstrated a high degree of misfit were less likely to be male, White, or diagnosed with AIDS but did not differ from the rest of the sample with respect to any other demographic or clinical characteristic listed in Table 2.

### 3.5. Person-Separation Reliability and Internal Consistency

The 20-item CES-D demonstrated acceptable person-separation reliability (index = 2.04) according to the set criterion of >2.0. The 15-item version had slightly lower person-separation reliability (index = 1.90), which did not reach the set criterion. Both versions were able to distinguish approximately 3 levels of depressive symptom severity. Cronbach's alpha coefficients for both the 20- and 15-item versions of the CES-D were acceptable (0.88 for both versions) and exceeded the set criterion of >0.80. Even with a 25% reduction of items (5/20), the 15-item version demonstrated similar reliability coefficients to the 20-item version.

### 3.6. Differential Item Functioning (DIF)

Finally, we analyzed the presence of DIF in relation to sociodemographic and clinical variables. Of the original 20 items, there was significant DIF on specific items in relation to gender, race, antidepressant use, and AIDS diagnosis (see Tables 3 and 5). For DIF by gender, item 17 (*crying*) was more easily endorsed by female and transgender participants compared to male participants (i.e., females and transgender participants had higher scores on this item than what would be expected by the Rasch model, while males had lower scores than expected), and item 15 (*unfriendly*) was more easily endorsed by transgender compared to male participants. For DIF by race, items 20 (*could not get going*), 18 (*sad*), and 6 (*depressed*) were more easily endorsed by White participants compared to Black participants, whereas item 19 (*felt disliked*) was more easily endorsed by Black participants than White participants. In addition, item 16 (*enjoyed life*) was more easily endorsed by White participants than by participants in the Other race group.

For DIF by antidepressant use, item 20 (*could not get going*) was more easily endorsed by those taking an antidepressant than by those who were not. Finally, for DIF by AIDS diagnosis, item 8 (*hopeful*) was more easily endorsed by those who had not been diagnosed with AIDS than by those who had. Somewhat fewer but similar patterns of DIF were evident in the 15-item version of the CES-D (see Table 5). As shown in Table 3, two of the items with DIF in the original 20-item scale (items 8 and 16) also demonstrated poor item fit and were not included in the 15-item version, thus accounting for some of the DIF differences between the 15-item and 20-item versions. There was no DIF related to age, education, income, or illicit drug use in either version.

### 3.7. Differential Test Functioning

To evaluate the impact of eliminating the five misfitting items, individual measures from the original 20-item CES-D and the 15-item version were compared by calculating the *z*-score of the difference between the scores. As only 6 participants (1.7%) demonstrated *z*-values exceeding ±1.96, we concluded that the 15-item version generates similar measures to the original CES-D for the majority of the sample, despite the lack of unidimensionality in both versions. Furthermore, the original 20-item CES-D and the 15-item measures were highly correlated (*r* = 0.93, *p* < 0.01), exceeding the criterion of >0.80 and *p* < 0.05.

### 3.8. Evaluation of the 10-Item Version [41]

In our final step, we also evaluated the 10-item version suggested by Zhang et al. [41] for use with adults living with HIV/AIDS (see Table 1). Even though the rating scale met the criteria in this version and differential test functioning was acceptable, one item (item #8) demonstrated misfit (10%) to the Rasch model, and the 10-item version also failed to meet the criteria for unidimensionality, as only 34.2% of the total variance in CES-D scores was explained by the first principal component. In addition, a higher-than-expected proportion of the sample (11.0%) demonstrated misfitting responses in this version. Most importantly, the 10-item version was not able to distinguish the sample into distinct levels of depressive symptom severity (separation index = 1.42).

## 4. Discussion

To our knowledge, this is the first psychometric evaluation of the CES-D using Rasch analysis in a sample of adults living with HIV/AIDS. The findings of this study indicate that there may be serious psychometric limitations to using the CES-D with this population. Five of the original 20 items demonstrate substantial item misfit to the Rasch model, but exclusion of these items resulted in little improvement of the scale's psychometric properties. In fact, both short forms we evaluated, one of which excluded all five of the misfitting items, demonstrated similar psychometric limitations to the full scale. Nonetheless, we recognize that the CES-D is widely used and its use will likely continue until an instrument with more robust psychometric properties is available. While there are other depression measures currently in use, to our knowledge, none have demonstrated robust psychometric properties for assessing depressive symptoms among adults with HIV/AIDS or other chronic illnesses. Thus, for those who use the CES-D to assess depressive symptoms among adults with HIV/AIDS, it is important that its psychometric limitations be considered, particularly when used in research settings and when comparing scores across groups, to avoid drawing invalid conclusions. Furthermore, we recommend that the 5 misfitting items be interpreted with particular caution, especially when their responses seem inconsistent with the rest of the scale, as they may not be measuring the same construct as other items.

Of the five misfitting items, three were positive affect items, which have been identified in other studies as being poorly correlated with the rest of the scale [34, 35] and not useful for distinguishing depressed and nondepressed adults with HIV disease [29]. While it is possible that the positive affect items represent a separate construct, given the reversed scaling of these items, the possibility of response bias should also be considered. The other two misfitting items (*poor appetite* and* restless sleep*) are somatic in nature. These symptoms are also relatively common among adults with HIV/AIDS [48] and may be associated with aspects of HIV disease or chronic illness that are unrelated to depression.

The results of this study raise concerns about the unidimensionality of the construct measured by the CES-D. Even the subscales representing Beck's four components of depression failed to meet the standards of unidimensionality as defined for our study sample, thereby raising questions about the measure's structural validity. Studies in other populations have also reported a lack of unidimensionality due to misfitting items, although the issue could generally be corrected by excluding the misfitting items [24, 30]. These results suggest that factors other than depressive symptoms might be influencing CES-D scores in this sample of adults with HIV/AIDS.

The gender-related DIF identified in this study was similar to that reported in non-HIV samples, with women generally being more likely to report* crying* and Blacks being more likely than Whites to report* feeling disliked* and less likely to report feeling* sad* or* depressed *or that they* could not get going*, regardless of their underlying level of depression [21, 27, 57–59]. This study also included a small sample of transgender adults, and several items (*crying*,* people were unfriendly*, and* lonely*) demonstrated DIF for this understudied group as well. Two clinical variables, antidepressant use and AIDS diagnosis, each demonstrated DIF on a single item in the 20-item CES-D. Somewhat unexpectedly, there was no DIF related to illicit drug use, despite higher CES-D scores among those who tested positive for illicit drugs compared to those who did not. It may be reassuring to know that drug use did not compromise the validity of CES-D scores in this sample, as conducting urine tests is not always feasible.

In light of the number of items with poor fit to the Rasch model or demonstrating DIF, the appropriateness of the clinical cutoff warrants further evaluation in this population. Items that are poorly correlated with the rest of the scale and items that result in differential responding in various subgroups may cause scores to vary systematically across subgroup, and it would be important to determine whether those differences are clinically meaningful with respect to clinical diagnosis and whether a higher or lower cutoff may be appropriate for different subpopulations of adults with HIV/AIDS. Further research in this area is warranted if the CES-D is to be used as a valid screening tool for clinical depression among a diverse population of adults living with HIV/AIDS.

Although it might be recommended that the five misfitting items identified in this study be omitted from the CES-D when used to assess depressive symptoms in adults living with HIV/AIDS, the resulting 15-item version was only slightly better in terms of unidimensionality, person-response validity, and DIF and was slightly worse with respect to person-separation reliability. Therefore, the psychometric properties of the 15-item version are not sufficiently better than those of the original CES-D to warrant such a recommendation.

A number of CES-D short forms have already been developed for use with various populations, either to minimize participant burden [60, 61] or to eliminate items that discriminate poorly between depressed and nondepression samples [62]. A 10-item version of the CES-D has been previously recommended for use among adults with HIV/AIDS [41]. However, to our knowledge, it has only been evaluated in relation to its sensitivity and specificity to a 20-item CES-D score ≥16, and aspects of its internal scale validity (item fit or DIF) were not assessed. Thus, our findings provide additional evidence of the psychometric properties of this version of the CES-D in a similar sample of adults with HIV/AIDS. Even though the 10-item version omits three of the five misfitting items identified in our current study, as well as five of the eight items demonstrating DIF, the criteria for item fit, unidimensionality, and person fit were not met. Perhaps the most problematic finding was that the 10-item CES-D version was unable to separate the sample into even two distinct levels of depressive symptom severity (as would be indicated if the separation index was at least 1.5 [46]), as this raises serious concern regarding this short form as a useful measure of depressive symptoms among adults with HIV/AIDS.

The findings of this study need to be considered in light of several limitations. First, information was not available regarding the participant's diagnostic status for depression other than the proxy of taking antidepressant medication, and therefore, the utility of individual items for distinguishing depressed from nondepressed respondents in this sample could not be determined. This study evaluated a number of demographic and clinical variables but was not large enough to examine DIF within groups, such as gender with differential effects by age or racial/ethnic group. Furthermore, the sample in this study was sociodemographically diverse but had insufficient numbers of Hispanic/Latino participants to specifically evaluate DIF for this group. Lastly, most of the adults in this sample had been diagnosed with HIV for many years, and therefore, our findings cannot be generalized to adults who have been newly diagnosed.

## 5. Conclusions

In conclusion, in our sample of adults living with HIV/AIDS, the CES-D lacked internal scale validity (i.e., unidimensionality), even after excluding items with poor fit to the Rasch model, and several items demonstrated significant DIF in relation to gender and race. In light of these issues, CES-D scores should be interpreted with caution in this and possibly other chronic illness populations. Further research is needed to determine appropriate clinical cutoff scores and identify brief measures of depression with better psychometric properties.

## Figures and Tables

**Table 1:** Overview of the analytic process using a Rasch model approach.

Step	Psychometric property	Statistical approach and criteria	Results		
			Original 20-item CES-D	Reduced 15-item CES-D (omits items with poor fit)^a^	Zhang et al. 10-item CES-D [41]
1	*Rating scale functioning*: does the rating scale function consistently across items? (substantive validity)	(i) Average measures for each step category and threshold on each item should advance monotonically (ii) *z*-values < 2.0 in outfit mean square *(MnSq)* values for step category calibrations [42]	Rating scale met criteria	Rating scale met criteria	Rating scale met criteria

2	*Internal scale validity*: how well do the actual item responses match the expected responses from the Rasch model? (content validity)	Item goodness-of-fit statistics *MnSq *values < 1.3 [43]	5 items failed to meet criterion^b^: (i) Item 4: *MnSq* = 1.58 (ii) Item 8: *MnSq* = 1.47 (iii) Item 11: *MnSq* = 1.33 (iv) Item 2: *MnSq* = 1.36 (v) Item 16: *MnSq* = 1.36	All items met criterion	One item failed to meet the criterion^b^: item 8: *MnSq* = 1.53

3	*Internal scale validity*: is the scale unidimensional (i.e., does it measure a single construct)? (structural validity)	Principal component analysis (i) ≥50% of total variance explained by first component (depressive symptoms) [44] (ii) Any additional component explains < 5% of the remaining variance after removing first component [44]	(i) First component explained 32.5% of total variance (ii) Second component explained 9.4% of total variance	(i) First component explained 37.9% of total variance (ii) Second component explained 7.4% of total variance	(i) First component explained 34.2% of total variance (ii) Second component explained 12.0% of total variance

4	*Person-response validity*: how well do the individual responses match expected responses from the Rasch model? (substantive validity)	Person goodness-of-fit statistics (i) *MnSq* values < 1.5 and *z-*value ≤ 2.0 (ii) ≤5% of sample fails to demonstrate acceptable goodness-of-fit values [45]	52 respondents (15.0% of sample) failed to demonstrate acceptable goodness-of-fit values	36 respondents (10.3% of sample) failed to demonstrate acceptable goodness-of-fit values	38 respondents (11.0% of sample) failed to demonstrate acceptable goodness-of-fit values

5	*Person-separation reliability*: can the scale distinguish ≥3 distinct groups of depression in the sample tested? (reliability)	Person-separation index ≥2.0 [46]	2.04	1.90	1.42

6	*Internal consistency*: are item responses consistent with each other? (reliability)	Cronbach's alpha coefficient >0.80 [46]	0.88	0.88	0.78

7	*Differential item functioning (DIF)*: are item difficulty calibrations stable in relation to key demographic variables? (generalizability validity)	Mantel-Haenszel statistic (i) *p* < 0.01 with Bonferroni correction [47] (ii) 1 item with DIF out of 20 may occur by chance and is deemed acceptable	Items with DIF (i) Gender: 15 & 17 (ii) Race: 20, 19, 18, 16 & 6 (iii) Antidepressant use: 20 (iv) AIDS diagnosis: 8	Items with DIF (i) Gender: 14 & 17 (ii) Race: 20, 19, 18 & 6	Not evaluated

8	*Differential test functioning (DTF)*: how consistent are the scores for the original CES-D and reduced-item scales?	(i) ≤5% of *z*-scores of the differences between the two test scores exceed ±1.96 (ii) Pearson correlation *r* > 0.80 and *p* < 0.05	Not applicable	(i) 6 measures (1.7%) had *z*-scores exceeding ±1.96 (ii) *r* = 0.933, *p* < 0.01	(i) 1 measure had a *z*-score exceeding ±1.96 (ii) *r* = 0.942, *p* < 0.01

*Note*. After initial evaluation of the original 20-item CES-D, a stepwise process was used whereby items failing to meet criteria were removed one at a time, and only those meeting criteria in earlier steps advanced to subsequent steps. If more than one item failed to meet a criterion, the item with the worst fit was removed and the step was repeated with the remaining items. The last column includes ^a^15-item version omitting misfitting items 2 (*appetite*), 4 (*as good as others*), 8 (*hopeful*), 11 (*restless sleep*), and 16 (*enjoyed life*).

^b^The five misfitting items did not all demonstrate misfit in the first iteration; some emerged in subsequent iterations; items are listed in the order of removal and the *MnSq* values shown reflect the iteration prior to the item's removal.

**Table 2:** CES-D scores by demographic and clinical characteristics, *M* (SD).

	*n*	Total CES-D score^a^	Positive affect^b^	Negative affect^a^	Somatic symptoms^a^	Interpersonal difficulty^a^
Full sample	347	17.2 (10.4)	8.2 (3.2)	5.5 (4.6)	6.7 (4.2)	1.2 (1.5)
Age in years						
Mean (SD): 45.1 (8.3)						
Range: 22–77 years						
<45 years	154	18.1 (10.2)	8.0 (3.2)	5.9 (4.6)	6.8 (4.0)	1.4 (1.5)^c^
≥45 years	193	16.5 (10.5)	8.4 (3.2)	5.1 (4.6)	6.7 (4.3)	1.1 (1.4)^c^
Gender						
Male	231	16.2 (10.3)^c^	8.4 (3.2)	5.2 (4.4)	6.4 (4.0)	1.1 (1.3)^c^
Female	93	19.4 (10.7)^c^	7.8 (3.3)	6.2 (5.1)	7.6 (4.6)	1.4 (1.6)
Transgender	23	18.1 (9.0)	7.7 (2.9)	5.4 (4.2)	6.6 (3.9)	2.0 (1.8)^c^
Race						
Black	140	16.5 (9.6)	8.1 (3.4)	4.8 (4.4)^c^	6.4 (4.3)	1.4 (1.5)^c^
White	140	16.7 (10.2)	8.4 (3.1)	5.7 (4.5)	6.5 (3.9)	1.0 (1.2)^c^
Other	67	19.7 (11.9)	8.0 (3.0)	6.5 (5.0)^c^	7.8 (4.5)	1.5 (1.7)
Education completed						
<High school	56	21.2 (11.4)^d^	7.6 (3.2)	6.9 (4.9)^c^	8.1 (4.8)	1.8 (1.8)^d^
High school	218	16.4 (9.8)^d^	8.3 (3.2)	5.1 (4.5)^c^	6.4 (4.0)	1.2 (1.4)^d^
College	73	16.6 (10.5)^d^	8.5 (3.2)	5.4 (4.7)	6.8 (3.9)	1.0 (1.3)^d^
Income ($/month)						
<$1,000	246	17.3 (10.4)	8.2 (3.2)	5.5 (4.6)	6.7 (4.3)	1.3 (1.5)
≥$1,000	101	16.9 (10.4)	8.3 (3.3)	5.4 (4.6)	6.8 (3.9)	1.0 (1.4)
CD4+ T-cell count	329^e^					
<200 (cells/mm^3^)	59	18.4 (11.0)	8.3 (3.0)	5.7 (4.6)	7.3 (4.2)	1.7 (1.7)^c^
≥200 (cells/mm^3^)	270	16.8 (10.3)	8.2 (3.3)	5.3 (4.6)	6.5 (4.2)	1.2 (1.5)^c^
Viral load (copies/mL)	320^e^					
<10,000	253	16.1 (10.1)^c^	8.4 (3.2)	5.0 (4.4)^c^	6.3 (4.0)^c^	1.1 (1.4)^c^
≥10,000	67	20.0 (11.1)^c^	7.9 (3.2)	6.7 (4.9)^c^	7.6 (4.6)^c^	1.7 (1.7)^c^
Years since HIV diagnosis						
<5	60	17.8 (11.3)	8.0 (3.3)	5.8 (5.3)	6.6 (4.7)	1.3 (1.7)
≥5	287	17.1 (10.2)	8.3 (3.2)	5.4 (4.5)	6.7 (4.1)	1.2 (1.4)
AIDS diagnosis						
No	170	17.9 (10.6)	7.9 (3.4)	5.7 (4.8)	6.8 (4.3)	1.3 (1.6)
Yes	177	16.5 (10.2)	8.6 (2.9)	5.2 (4.4)	6.6 (4.0)	1.2 (1.4)
Antiretroviral therapy?						
No	102	18.0 (10.6)	8.0 (3.3)	6.1 (4.8)	6.5 (4.3)	1.4 (1.7)
Yes	245	16.9 (10.3)	8.3 (3.2)	5.2 (4.5)	6.8 (4.1)	1.2 (1.3)
Taking antidepressant?						
No	209	16.1 (10.0)^c^	8.2 (3.2)	4.9 (4.4)^c^	6.3 (4.0)^c^	1.2 (1.4)
Yes	138	18.8 (10.8)^c^	8.2 (3.2)	6.3 (4.9)^c^	7.4 (4.4)^c^	1.4 (1.6)
Illicit drug test result	346^e^					
Negative	316	16.9 (10.4)^c^	8.4 (3.2)^c^	5.4 (4.6)	6.7 (4.2)	1.2 (1.4)^c^
Positive	30	20.9 (9.0)^c^	6.7 (3.3)^c^	6.5 (4.9)	7.3 (4.2)	1.8 (1.7)^c^

*Note*. Nonparametric tests (Kruskal-Wallis and Mann-Whitney *U*) were used to compare the nonnormal distributions of CES-D scores across demographic and clinical groups.

^a^Higher scores indicate fewer and/or less frequent depressive symptoms; total CES-D scores ≥ 16 indicate need to seek clinical evaluation for depression.

^b^Not reverse coded; higher scores indicate more positive affect.

^c^Difference between the groups was significant (*p* < 0.05).

^d^Adults who did not complete high school differed from the other two groups (*p* < 0.05).

^e^Smaller sample due to missing laboratory or drug test data.

**Table 3:** CES-D items demonstrating poor fit or differential item function.

	Poor item fit	DIF
(1) *I was bothered by things that usually don't bother me.*		
(2) I did not feel like eating; my appetite was poor.	X	
(3) *I felt that I could not shake off the blues even with help from my family or friends.*		
(4) I felt I was just as good as other people.	X	
(5) *I had trouble keeping my mind on what I was doing.*		
(6) I felt depressed.		Race
(7) *I felt that everything I did was an effort.*		
(8) I felt hopeful about the future.	X	AIDS
(9) *I thought my life had been a failure.*		
(10) *I felt fearful.*		
(11) My sleep was restless.	X	
(12) *I was happy.*		
(13) *I talked less than usual.*		
(14) *I felt lonely.*		
(15) People were unfriendly.		Gender
(16) I enjoyed life.	X	Race
(17) I had crying spells.		Gender
(18) I felt sad.		Race
(19) I felt that people dislike me.		Race
(20) I could not get going.		Race

*Note*. Italicized items were not biased by misfit or differential item function (DIF).

**Table 4:** Variance explained in each CES-D subscale.

Subscale	Items included in each subscale	Variance explained
Positive affect	4, 8, 12, 16	44.2%
Negative affect	3, 6, 9, 10, 14, 17, 18	47.6%
Somatic symptoms and retarded activity	1, 2, 5, 7, 11, 13, 20	37.7%
Interpersonal difficulties	15, 19	46.2%

**Table 5:** Differential item functioning in the original 20-item CES-D and a 15-item version.

Demographic and clinical variables	Original 20-item version	15-item version (omitting items 2, 4, 8, 11 & 16)
Gender (male, female, and transgender)	Item 15: *unfriendly* Transgender^*∗*^ > male Item 17: *crying* Female, transgender^*∗*^ > male	Item 14: *lonely* Males > transgender^*∗*^ Item 17: *crying* Female, transgender^*∗*^ > male

Race (Black, White, and Other)	Item 20: *could not get going* White > Black Item 19: *felt disliked* Black > White Item 18: *sad* White > Black Item 16: *enjoyed life* White > Other race Item 6: *depressed* White > Black	Item 20: *could not get going* White > Black Item 19: *felt disliked* Black > White Item 18: *sad* White > Black Item 6: *depressed* White > Black (Item 16 excluded)

Antidepressant use (No/Yes)	Item 20: *could not get going* Yes > No	None

AIDS diagnosis (No/Yes)	Item 8: *hopeful* No AIDS > AIDS	None (item 8 excluded)

*Note*. > indicates that the item was more easily endorsed by the first group than the second group. There was no differential item function related to age, education, income, duration of HIV diagnosis, or illicit drug use.

^*∗*^The transgender group was small (*n* = 23) and results should thus be interpreted with caution.
